# Metabolic Syndrome and Dental Caries Among Adults in the United States: A Cross-Sectional Study From the National Health and Nutrition Examination Survey, 2015-2018

**DOI:** 10.7759/cureus.95397

**Published:** 2025-10-25

**Authors:** Alhassan Hameedaldeen, Zuhair S Natto, Hesham Alhazmi, Catherine Hayes

**Affiliations:** 1 Dental Public Health, King Abdulaziz University, Jeddah, SAU; 2 Oral Health Policy and Epidemiology, Harvard School of Dental Medicine, Boston, USA; 3 Pediatric Dentistry, Umm Al-Qura University, Makkah, SAU

**Keywords:** dental caries, hdl, hypertension, metabolic syndrome, triglycerides, type 2 diabetes mellitus

## Abstract

Introduction: Metabolic syndrome (MetS) is a cluster of conditions that increases the risk of cardiovascular disease and diabetes. It has become a major global health concern, significantly affecting adults worldwide. Dental caries is also one of the most prevalent diseases globally, sharing common risk factors with MetS. Despite emerging evidence suggesting a link between the two conditions, findings remain inconsistent, and no study has explored this association in a nationally representative US sample. Our study aimed to assess the association between dental caries and MetS, as well as to evaluate the effect of the five components of MetS, both separately and collectively, on dental caries.

Methods: In this cross-sectional study, data from the National Health and Nutrition Examination Survey (NHANES 2015-2018) were used to draw a sample of adults aged 30 years and older who had completed laboratory and clinical examinations for both MetS and dental caries. Dental caries outcomes were assessed using untreated caries and the decayed, missing, and filled teeth (DMFT) index, analyzed both as discrete and categorical (very low, low, moderate, and high) variables. MetS was defined using the National Cholesterol Education Program’s Adult Treatment Panel III (NCEP-ATP III) criteria, requiring at least three of the five MetS components. Covariates included demographics, socioeconomic status, smoking, sugar intake, and dental visit history. Weighted descriptive statistics were calculated to include prevalence, chi-square, and p-value. Also, logistic regression was used to estimate crude odds ratios (cOR), adjusted odds ratios (aOR), and 95% confidence intervals (CI) for associations between untreated caries and MetS prevalence, number of MetS components, and individual components. Negative binomial regression was used to estimate crude mean ratios (cMR), adjusted mean ratios (aMR), and 95% CIs for associations with DMFT.

Results: There were 3,291 participants who met our inclusion criteria. Approximately 1,342 participants (41%) had MetS, where the most affected groups were age 70 and above (267 participants, 45.13%), Hispanic race (442 participants, 44.47%), untreated dental caries (408 participants, 46.31%), and high DMFT score (588 participants, 44.80%) (p-value < 0.05). The odds of having untreated caries and a high DMFT score among the MetS group compared to no MetS increased by 34% (95% CI: 1.03-1.75) and 10% (95% CI: 1.11-1.23), respectively. Having all five MetS components increased the chance of having a higher DMFT score by 17% compared to having no MetS, but there was no statistically significant association between the number of MetS components and untreated caries. When one of the MetS components was insulin resistance, abdominal obesity, or low high-density lipoprotein (HDL), the odds of having untreated caries were significantly higher by 40% (95% CI: 1.06-1.86), 40% (95% CI: 1.08-1.83), and 45% (95% CI: 1.04-2.04), respectively.

Conclusions: Our study suggests that individuals with MetS are more likely to have dental caries. These findings highlight the importance of developing targeted prevention and management guidelines for dental caries in patients with MetS. The results can guide health providers in educating patients about MetS components to improve control and lower dental caries risk. Future cohort studies are needed to establish temporality and clarify the causal relationship between MetS and dental caries.

## Introduction

Over the past twenty years, metabolic syndrome (MetS) has become widely recognized as a major health issue affecting all age groups [1]. The prevalence among adults is about 30% in most countries, raising serious global public health concerns [2]. The term "MetS" was first introduced by Gerald Reaven in 1988, and the disorder is also referred to as insulin resistance syndrome or syndrome X [3]. It is defined as a cluster of conditions that increase the risk of cardiovascular disease and type 2 diabetes, including insulin resistance, dyslipidemia (high triglycerides and low high-density lipoprotein (HDL)), hypertension, and central obesity [4]. Several institutions have defined MetS with the consensus that it involves having at least three out of five specific components. However, they differ in their criteria regarding which three components were required and the thresholds for each component [5]. While the precise pathological mechanisms are not yet fully understood, previous studies indicate that chronic subclinical inflammation is linked to metabolic dysfunction, including MetS [6]. The association between MetS and several diseases, including oral diseases, has been widely studied [1].

Dental caries is recognized as one of the most widespread diseases globally, resulting in significant health challenges and economic impacts [7]. Dental caries is defined by the European Organization for Caries Research (ORCA) and the International Association for Dental Research (IADR) Cariology Research Group as “a biofilm-mediated, diet-modulated, multifactorial, non-communicable, dynamic disease resulting in net mineral loss of dental hard tissues. It is determined by biological, behavioral, psychosocial, and environmental factors. As a consequence of this process, a carious lesion develops" [8]. Few studies have shown that dental caries and MetS are associated, sharing common risk factors such as abdominal obesity, high sugar intake, body weight, age, smoking, alcohol use, income level, and lack of physical activity [9]. Moreover, some studies suggested that they are interrelated because both are associated with chronic low-grade inﬂammation and insulin resistance [10].

A limited number of studies have investigated the relationship between dental caries and MetS, mainly in Asian countries like Japan, China, and Iran [1,2,6]. In addition, the literature has shown controversy regarding the association between dental caries and MetS [1]. To our knowledge, no published article used a nationally representative sample of the US population to study the association between dental caries and MetS. The aim of this study is to assess the association between MetS and dental caries using the National Health and Nutrition Examination Survey (NHANES) dataset, a nationally representative sample of the US population.

## Materials and methods

Study population

This study was conducted by the National Center for Health Statistics (NCHS), Centers for Disease Control and Prevention (CDC). The NHANES data provide information on the health and nutritional status of noninstitutionalized civilian residents (all ages) of all states and the District of Columbia. It is a cross-sectional dataset that consists of personal interviews and a standardized physical examination, which includes the measurement of general health status, clinical examination, and laboratory testing (blood and urine specimens) performed in a mobile examination center [11]. This study analyzed data from adults aged 30 and older who participated in the NHANES 2015-2018 in the United States. A total of 3,291 individuals with available blood test results and dental caries examination history were included in the analysis. Although we used publicly available secondary data (not considered human subjects research), a Harvard Institutional Review Board (IRB) exemption (IRB23-1407) was obtained. This cross-sectional study was carried out in compliance with the guidelines of the Strengthening the Reporting of Observational Studies in Epidemiology (STROBE) statement.

Dental caries measurement

First, we used untreated dental caries on adults’ permanent dentition. We categorized having untreated dental caries as “yes” or “no,” where yes is given to participants who have at least one dental caries present on any tooth and no if there is no caries present (white spot lesions are categorized as no). Second, we used decayed, missing, and filled teeth (DMFT) as a discrete variable and as a categorical variable categorizing the total dental caries experience as very low (having less than five carious teeth), low (from five to 8.9 carious teeth), moderate (from nine to 13.9 carious teeth), and high (more than 13.9 carious teeth) [12]. The DMFT variable was also treated as a discrete variable when the analysis was performed.

Metabolic syndrome definition

In order for a participant to be considered a patient with MetS, we used the National Cholesterol Education Program’s Adult Treatment Panel III (NCEP-ATP3) diagnostic criteria report, where three or more of the following five MetS components have to be met. A waist circumference greater than 40 inches in men or 35 inches in women is one criterion. Another is having a blood pressure reading higher than 130/85 mmHg. Elevated fasting triglyceride levels, specifically above 150 mg/dl, also count toward the diagnosis. In addition, a low fasting HDL cholesterol level-less than 40 mg/dl in men or 50 mg/dl in women-is considered a risk factor. Finally, a fasting blood sugar level exceeding 100 mg/dl meets the last of the five possible criteria [13].

Covariates

Secondary variables included demographic characteristics (age, gender, race, poverty-income ratio (PIR), and education level), last dental visit, smoking status, and sugar consumption.

Age was categorized into three groups: 30-49, 50-69, and 70 and older. Regarding the race/ethnicity variable, it was classified as White, African American, Hispanic, Asian, and Other individuals. "Others” under race includes participants who self-identified as not being categorized as Mexican American, Other Hispanic, Non-Hispanic White, Non-Hispanic Black, or Non-Hispanic Asian individuals. They self-identified as non-Hispanic multiracial individuals. The participants were divided into three groups according to PIR, which are less than 1.38 PIR (low income), from 1.38 to 3.99 (middle income), and more than 3.99 PIR (high income). Education level was divided into four groups as follows: less than high school, high school, some college, college or higher.

Regarding the last dental visit, participants were divided into adults who had their last dental visit within one year and adults who had their last dental visit more than one year ago. Smoking status was used to categorize participants into current smokers (who smoked more than 100 cigarettes in their life, and they still smoke), former smokers (who smoked more than 100 cigarettes in their life, but they do not smoke now), and never smokers (who did not smoke more than 100 cigarettes in their life) [14]. Finally, based on the United Kingdom National Health Service, participants who consume more than 90 g per day were considered as high sugar consumers, and those who consume 90 g or less were in the low sugar consumption group [15].

Statistical analysis

Data were analyzed using Stata software version 16, USA (StataCorp, USA). Descriptive analyses were conducted to understand the characteristics of our population. It included the weighted prevalence and chi-square p-value of demographics, last dental visit, smoking status, sugar consumption, untreated dental caries, DMFT, and MetS.

Univariable and multivariable regression analyses were performed using forward selection, with confounding covariates added sequentially to the model. Variables that improved the adjusted R² were retained in the final model. All regression analyses accounted for the complex survey design of NHANES by incorporating appropriate sampling weights, strata, and primary sampling units. Three logistic regression analyses were conducted to estimate the crude odds ratio (cOR), adjusted odds ratio (aOR), and 95% CI of the association between untreated dental caries and the three variables separately: MetS prevalence, MetS number of components, and MetS different components. Also, negative binomial regression analyses were conducted to estimate the crude mean ratio (cMR), adjusted mean ratio (aMR) and 95% CI of the association between DMFT and the same three variables related to MetS.

## Results

Demographic characteristics

Table 1 shows the characteristics of US adults with MetS. In this study, there were 3,291 participants who had completed their laboratory and physical exam, which is required for MetS classification. Approximately 41% (1,342 participants) had MetS, where it was equally distributed between both sexes. Participants over the age of 70 were the highest to suffer from MetS, with 267 participants (45.13%), while those between the ages of 30 and 49 were the lowest, with 415 participants (32.14%). The highest MetS prevalence was among the Hispanic race, 442 participants (44.47%), while the lowest prevalence was among the Asian race, 122 participants (27.80%). Less than a high school education level and low-income level had a higher prevalence of MetS of 320 participants (43.51%) and 371 participants (44.70%), respectively. Moreover, never smokers were the lowest to get MetS, with 711 participants (34.94%) compared to former smokers with 399 participants (45.61%) and current smokers with 230 participants (39.84%). Sugar consumption did not increase the risk of having MetS, while having a dental visit more than one year ago had a higher MetS prevalence of 637 participants (43.64%). Finally, MetS prevalence was higher among those with untreated dental caries (408 participants, 46.31%) and high DMFT scores (588 participants, 44.80%).

**Table 1:** Characteristics of adults in the US with metabolic syndrome, NHANES 2015–2018 MetS: metabolic syndrome; DMFT: decayed, missing, and filled teeth; NHANES: National Health and Nutrition Examination Survey a: weighted percentages; b: weighted chi-square; c*: *p-value of the weighted chi-square test; *: significant at α <0.05

Category	Total Frequency (%^a^)	MetS Frequency (%^a^)	Chi-square^b^	P-value^c^
Sex				
Male	1607 (49.10)	635 (39.62)	0.41	0.527
Female	1684 (50.90)	707 (38.02)		
Age groups				
30-49	1301 (43.14)	415 (32.14)	12.95	<0.001*
50-69	1388 (43.99)	636 (43.86)		
70+	547 (12.87)	267 (45.13)		
Race				
White	1108 (66.11)	466 (38.94)	4.26	0.013*
Hispanic	919 (14.51)	442 (44.47)		
Black	689 (9.71)	251 (35.23)		
Asian	437 (5.40)	122 (27.80)		
Other	138 (4.27)	61 (39.34)		
Education level				
Less than high school	693 (12.23)	320 (43.51)	3.18	0.048*
High school	716 (22.93)	299 (42.79)		
Some college	1003 (31.22)	428 (40.37)		
College or higher	879 (33.62)	295 (32.91)		
Income level				
Low income	841 (17.69)	371 (44.70)	9.40	<0.001*
Middle income	1245 (39.59)	539 (43.07)		
High income	835 (42.72)	283 (33.33)		
Smoking status				
Never smokers	1867 (55.13)	711 (34.94)	8.09	<0.001*
Former smokers	848 (28.77)	399 (45.61)		
Current smokers	573 (16.10)	230 (39.84)		
Sugar consumption				
Low sugar	1613 (47.56)	676 (39.61)	0.28	0.6009
High sugar	1530 (52.44)	614 (38.19)		
Last dental visit				
1 Year or less	1860 (62.67)	698 (35.86)	7.13	<0.001*
More than a year	1419 (37.33)	637 (43.64)		
Untreated caries				
No	1839 (72.76)	711 (36.27)	12.25	0.001*
Yes	909 (27.24)	408 (46.31)		
DMFT level				
Very low DMFT	587 (16.45)	204 (29.85)	9.46	<0.001*
Low DMFT	616 (19.65)	201 (32.22)		
Moderate DMFT	802 (27.43)	349 (40.92)		
High DMFT	1286 (36.47)	588 (44.80)		

Association between caries, MetS, and related factors

Table 2 presents univariable and multivariable logistic regression to assess the association between untreated caries with MetS and the covariates. Also, it presents negative binomial regression to assess the association between DMFT with MetS and the covariates. The odds of having untreated caries are significantly lower among females by 34% (95% CI: 0.47-0.92) compared to males after adjusting for confounders. Regarding age, both the crude and adjusted models showed no significant difference in having untreated caries between the different age groups. When comparing the White race to others, the crude odds of having untreated caries were higher among the Hispanic and Black races, but lower when compared to the Asian race (marginal significance). However, the aOR was only significant when comparing the White race to the Black race, with 63% (95% CI: 1.07-2.48) higher odds of having untreated caries among the Black race. The odds of having untreated caries are significantly lower among those with a college degree or higher by 63% (95% CI: 0.20-0.70) compared to those with a high school degree after adjusting for confounders. Similarly, the odds of having untreated caries are significantly lower among those with high income by 56% (95% CI: 0.26-0.72) compared to lower income. The aOR was significant when comparing never smokers to current smokers, where current smokers had 2.13 (95% CI: 1.48-3.06) times the odds of having untreated caries. The odds of having untreated caries were three times higher among those with more than a one-year dental visit compared to the less than one-year group, and 1.34 (95% CI: 1.03-1.75) times higher among the MetS group compared to the no MetS group.

**Table 2:** Univariable and multivariable regression analysis for the association between untreated caries and DMFT with MetS prevalence and the covariates OR: odds ratio; CI: 95% confidence interval; MetS: metabolic syndrome; DMFT: decayed, missing, and filled teeth *: significant at α <0.05

Category	Untreated Caries				DMFT				
	Crude OR(CI)	P-value	Adjust OR(CI)	P-value	Crude MR(CI)		P-value	Adjust MR(CI)	P-value
Sex (ref: male)									
Female	0.73 (0.64-0.85)	<0.001*	0.66 (0.47-0.92)	0.018*	1.08 (1.04-1.12)	<0.001*		1.10 (1.03-1.18)	0.096*
Age groups (ref: 30-49)									
50-69	0.98 (0.81-1.18)	0.808	1.04 (0.68-1.58)	0.843	1.64 (1.56-1.72)	<0.001*		1.72 (1.58-1.87)	<0.001*
70+	0.82 (0.63-1.06)	0.125	0.77 (0.47-1.24)	0.250	2.21 (2.11-2.32)	<0.001*		2.22 (2.03-2.42)	<0.001*
Race (ref: White)									
Hispanic	1.53(1.14-2.05)	0.006*	0.92 (0.58-1.45)	0.691	0.83 (0.79-0.88)	<0.001*		0.91 (0.83-0.99)	0.039*
Black	2.53 (2.00-3.20)	<0.001*	1.63 (1.07-2.48)	0.027*	0.89 (0.84-0.94)	<0.001*		0.93 (0.86-0.99)	0.041*
Asian	0.75 (0.56-1.00)	0.052	1.03 (0.56-1.89)	0.927	0.76 (0.69-0.84)	<0.001*		0.95 (0.86-1.06)	0.319
Other	1.49 (0.96-2.32)	0.074	0.66 (0.28-1.55)	0.311	0.95 (0.82-1.10)	0.481		0.82 (0.66-1.04	0.092
Education level (ref: less than high school)									
High school	0.85 (0.70-1.03)	0.087	0.93 (0.60-1.44)	0.719	1.11 (1.03-1.21)	0.010*		0.99 (0.88-1.10)	0.793
Some college	0.52 (0.44-0.61)	<0.001*	0.65 (0.41-1.02)	0.059	1.02 (0.95-1.10)	0.493		0.99 (0.88-1.12)	0.905
College or higher	0.17 (0.13-0.21)	<0.001*	0.37 (0.20-0.70)	0.005*	0.88 (0.81-0.95)	0.002*		0.88 (0.79-0.98)	0.021*
Income level (ref: low income)									
Middle income	0.53 (0.44-0.64)	<0.001*	0.79 (0.55-1.15)	0.195	1.01 (0.96-1.07)	0.575		0.98 (0.91-1.06)	0.660
High income	0.16 (0.13-0.20	<0.001*	0.44 (0.26-0.72)	0.004*	0.92 (0.86-0.97)	0.008*		0.89 (0.81-0.99)	0.027*
Smoking status (ref: never smokers)									
Former smokers	1.29 (1.05-1.59)	0.019*	0.87 (0.59-1.29)	0.462	1.22 (1.17-1.27)	<0.001*		1.13 (1.06-1.21)	0.001*
Current smokers	3.64 (2.98-4.45)	<0.001*	2.13 (1.48-3.06)	<0.001*	1.15 (1.09-1.21)	<0.001*		1.20 (1.11-1.30)	<0.001*
Sugar consumption (ref: low sugar)									
High sugar	1.23 (1.04-1.45)	0.015*	1.28 (0.92-1.76)	0.126	1.02 (0.98-1.06)	0.296		1.08 1.02-1.15)	0.018*
Last dental visit (ref: 1 year or less)									
More than a year	4.42 (3.57-5.46)	<0.001*	2.99 (2.18-4.10)	<0.001*	0.90 (0.86-0.94)	<0.001*		0.89 (0.84-0.95)	<0.001*
MetS (ref: no)									
Yes	1.52 (1.19-1.93)	0.002*	1.34 (1.03-1.75)	0.033*	1.17 (1.11-1.23)	<0.001*		1.10 (1.03-1.17)	0.004*

The second half of Table 2 shows the DMFT association with MetS and the covariates. Participants with high income and college or higher education had significantly lower mean DMFT scores compared to those with low income and less than high school education (aMR: 0.89 and 0.88, respectively; p-values: 0.027 and 0.021, respectively). Moreover, people older than 69 years had a DMFT score 2.2 times higher than those between 30 and 49. Racial differences were observed, with Black and Hispanic individuals demonstrating significantly lower mean scores of DMFT compared to White individuals (aMR: 0.93 and 0.91, respectively; P-values: 0.041 and 0.039, respectively). Current smokers had a 20% higher DMFT score compared to never smokers, and the high sugar consumption group had an 8% higher DMFT score. In contrast to untreated caries, females had a 10% higher DMFT score compared to males, and individuals whose last dental visit occurred more than one year ago had an 11% lower DMFT score compared to those who visited the dentist within the past year. Finally, participants with MetS exhibited significantly higher mean DMFT scores compared to those with no MetS (aMR: 1.10, p-value: 0.004).

Impact of different combined components

Table 3 presents univariable and multivariable logistic regression to assess the association between both untreated caries and the MetS number of components. Also, it presents negative binomial regression to assess the association between DMFT and MetS. Participants who had all five MetS components had the highest odds of 1.83 (with marginal significance, p-value = 0.052) for having untreated caries compared to the no MetS group. However, when adjusting for confounders, the odds of having untreated caries increased to 2.07 for those having the five components, but it was not statistically significant (p-value: 0.08). Moreover, individuals with all five MetS components had the highest crude and adjusted MR for having a higher DMFT score compared to the no MetS group (1.23 and 1.17, respectively; P-values: <0.001 and 0.004, respectively).

**Table 3:** Univariable and multivariable regression analysis for the association between untreated caries and DMFT with MetS number of components OR: odds ratio; CI: 95% confidence interval; MetS: metabolic syndrome; DMFT: decayed, missing, and filled teeth *: significant at α <0.05

Category	Untreated Caries				DMFT			
	Crude OR(CI)	P-value	Adjust OR(CI)	P-value	Crude MR(CI)	P-value	Adjust MR(CI)	P-value
MetS level (ref: no MetS)								
3 Components	1.56 (1.13-2.15)	0.009*	1.34 (0.92-1.95)	0.114	1.17 (1.11-1.24)	<0.001*	1.10 (1.03-1.19)	0.012*
4 Components	1.36 (1.03-1.79)	0.029*	1.20 (0.86-1.65)	0.250	1.15 (1.06-1.23)	<0.001*	1.07 (0.98-1.18)	0.130
5 Components	1.83 (0.99-3.37)	0.052	2.07 (0.90-4.75)	0.080	1.23 (1.13-1.35)	<0.001*	1.17 (1.06-1.28)	0.004*

Impact of different single components

Table 4 illustrates univariable and multivariable regression to assess the association between dental caries and the five MetS components separately. The regression analysis indicated that there was no significant association between dental caries and each MetS component separately, after adjusting for confounders. The only exception was for the positive association between DMFT and HDL (aMR: 1.10, P-value: 0.02).

**Table 4:** Univariable and multivariable regression analysis for the association between untreated caries and DMFT with each MetS component separately OR: odds ratio; CI: 95% confidence interval; MetS: metabolic syndrome; DMFT: decayed, missing, and filled teeth *: significant at α <0.05

Category	Untreated Caries				DMFT			
	Crude OR(CI)	P-value	Adjust OR(CI)	P-value	Crude MR(CI)	P-value	Adjust MR(CI)	P-value
Insulin resistance (ref: no)								
Yes	1.20 (0.94-1.53)	0.134	0.94 (0.67-1.329)	0.700	1.16 (1.10–1.24)	<0.001*	1.01 (0.93-1.09)	0.864
Abdominal obesity (ref: no)								
Yes	1.14 (0.96-1.34)	0.119	1.18 (0.79-1.77)	0.383	1.13 (1.09-1.18)	<0.001*	1.01 (0.94-1.08)	0.831
Hypertension (ref: no)								
Yes	1.44 (1.22-1.69)	<0.001*	1.03 (0.77-1.37)	0.830	1.20 (1.16-1.24)	<0.001*	1.02 (0.95-1.10)	0.505
High HDL (ref: no)								
Yes	1.51 (1.26-1.81)	<0.001*	1.41 (0.92-2.16)	0.099	1.03 (0.99-1.07)	0.171	1.10 (1.02-1.19)	0.020*
Low triglyceride (ref: no)								
Yes	1.24 (0.98-1.58)	0.075	0.96 (0.64-1.42)	0.802	1.04 (0.98-1.11)	0.186	1.00 (0.93-1.08)	0.995

Table 5 illustrates univariable and multivariable regression to assess the association between dental caries and the collective effect of MetS's different components. Participants with MetS and having insulin resistance as one of the components had 40% higher odds of having untreated caries compared to those with no MetS. Similarly, those with MetS and one of the components was abdominal obesity or low HDL had higher odds for having untreated caries compared to the no MetS group by 40 percent and 45 percent, respectively. On the other hand, although the odds of having untreated caries are also higher among those with MetS and having hypertension or high triglycerides as one of the components, it was not statistically significant.

**Table 5:** Univariable and multivariable regression analysis for the association between untreated caries and DMFT with each MetS component collectively OR: odds ratio; CI: 95% confidence interval; MetS: metabolic syndrome; DMFT: decayed, missing, and filled teeth *: significant at α <0.05

MetS components	Untreated Caries				DMFT			
	Crude OR(CI)	P-value	Adjust OR(CI)	P-value	Crude MR(CI)	P-value	Adjust MR(CI)	P-value
MetS with insulin resistance (ref: no MetS)								
Absent	1.24 (0.71-2.18)	0.434	0.87 (0.36-2.12)	0.741	1.00 (0.86-1.16)	0.996	1.11 (0.94-1.30)	0.189
Present	1.54 (1.19-1.99)	0.002*	1.40 (1.06-1.86)	0.023*	1.18 (1.13-1.24)	<0.001*	1.10 (1.03-1.17)	0.006*
MetS with abdominal obesity (ref: no MetS)								
Absent	1.91 (1.05-3.49)	0.035*	0.91 (0.40-2.10)	0.822	1.12 (0.99-1.28)	0.074	1.10 (0.97-1.25)	0.135
Present	1.48 (1.15-1.90)	0.004*	1.40 (1.08-1.83)	0.017*	1.17 (1.12-1.23)	<0.001*	1.10 (1.04-1.16)	0.004*
MetS with hypertension (ref: no MetS)								
Absent	1.73 (1.32-2.27)	<0.001*	1.60 (1.21-2.14)	0.004*	1.12 (1.03-1.22)	0.011*	1.12 (1.03-1.23)	0.014*
Present	1.39 (1.04-1.86)	0.026*	1.19 (0.84-1.67)	0.293	1.20 (1.14-2.25)	<0.001*	1.08 (1.01-1.16)	0.022*
MetS with low HDL (ref: no MetS)								
Absent	1.23 (0.84-1.81)	0.272	1.19 (0.75-1.88)	0.427	1.21 (1.14-1.27)	<0.001*	1.06 (0.98-1.14)	0.115
Present	1.75 (1.33-2.29)	<0.001*	1.45 (1.04-2.04)	0.032*	1.14 (1.07-1.22)	<0.001*	1.13 (1.05-1.21)	0.003*
MetS with high triglyceride (ref: no MetS)								
Absent	1.59 (1.14-2.21)	0.008*	1.35 (0.93-1.97)	0.104	1.20 (1.13-1.27)	<0.001*	1.10 (1.03-1.18)	0.008*
Present	1.45 (1.11-1.89)	0.008*	1.33 (0.96-1.86)	0.084	1.14 (1.07-1.22)	<0.001*	1.09 (1.03-1.17)	0.010*

Regarding the association between DMFT and MetS components, all five components (when assessed individually) showed a significant association with DMFT after adjusting for confounders. Similar to untreated caries, a higher mean DMFT score was mainly associated with having insulin resistance, abdominal obesity, or low HDL levels as one of the MetS components (aMR: 1.10, 1.10, 1.13, respectively; P-value: 0.006, 0.004, 0.003, respectively).

When comparing participants without MetS to those who had the three most significant MetS components (insulin resistance, abdominal obesity, and low HDL), the odds of having untreated caries were higher among participants having the three components (aOR: 1.56, P-value: 0.01). Similarly, the mean DMFT score is higher among participants having the three components (aMR: 1.13, P-value: 0.002).

## Discussion

In this study, the relationship of dental caries on MetS was evaluated using a large, nationally representative sample of 3,291 participants. MetS prevalence was higher among those having untreated dental caries and high caries experience, older than 69, Hispanic individuals, those with a low education level, those with a low income, smokers, and participants who missed their annual dental visit. Although it was reported in the literature that high sugar consumption is associated with both MetS and untreated caries [6,15,16], our study showed that there was no significant association. However, there was a statistically significant association between full dental caries experience (DMFT) and high sugar consumption (aMR: 1.08, p-value: 0.018). Moreover, the odds of having untreated caries were higher among males, Black individuals, those with low education levels, those with low income, smokers, and participants who missed their annual dental visit. In addition, the adjusted odds of having untreated caries were 34% higher among those with MetS compared to the no MetS group (aOR = 1.34, 95% CI 1.03-1.75, P-value = 0.033). Similarly, the aMR of having a higher DMFT score was significantly higher among the MetS group compared to the no MetS group (aMR = 1.10, 95% CI 1.03-1.17, p = 0.004).

After adjusting for confounding factors, the number of MetS components was not significantly associated with having untreated dental caries. In contrast, having all five MetS components showed the strongest statistically significant association with a higher DMFT mean score. Moreover, when the MetS components were assessed separately, none of the components were significantly associated with either untreated caries or DMFT, except for the positive association between HDL and the DMFT mean score. On the other hand, when the collective effect of the MetS components was assessed, insulin resistance, abdominal obesity, and low HDL had a significantly positive association with untreated caries as well as a higher DMFT mean score. Although the odds of having untreated caries were higher when hypertension was one of the components, the absence of hypertension showed a significantly stronger association. Thus, hypertension alone did not drive the association; rather, the observed effect may be confounded by the clustering of other stronger components. Similarly, although the MR of having a higher DMFT score was higher when hypertension or high triglyceride levels were one of the components, the absence of them showed a slightly stronger association.

A limited number of studies examined the association between dental caries and MetS, including its components [6]. One study that coincides with our results is Ojima et al., suggesting that dental caries were associated with MetS in early middle-aged Japanese men [2]. Also, a prospective study by Adachi et al. suggested that decayed teeth are associated with the development of MetS [9]. On the other hand, Esfanjani et al. indicated the opposite relationship, as dental caries and MetS had a negative association [17]. In addition, Song et al. indicated no association between dental caries and MetS [18]. The inconsistency with our results could be due to differences in the mean age of participants, differences in diagnostic criteria used for dental caries and MetS, inappropriate control of confounding factors, and the occurrence of selection bias.

This article has some limitations. It is a cross-sectional study, which limits the ability to determine temporality and prevents establishing causality. Moreover, the NHANES dataset does not include data from institutionalized individuals, restricting our ability to explore the relationship between dental caries and MetS prevalence in vulnerable institutionalized groups [11]. Also, NHANES does not provide radiographs or radiographic findings for a better assessment of dental caries [11]. In addition, the control of important factors influencing the development of dental caries, such as frequency of oral hygiene practices (tooth brushing and flossing) and the use of fluoridated toothpaste, was not reported in this study population.

However, this study has several strengths. NHANES provides a nationally representative sample of the US population, minimizing selection bias. The collected data about dental caries and MetS are from laboratory tests and clinical examinations done by professionals, not reported by participants [11]. Furthermore, this study accounted for known confounding factors, including sugar consumption, smoking, last dental visit, and various demographic variables. This analysis offers a novel opportunity to understand the association between dental caries and MetS, helping to shape future research directions.

The evidence linking dental caries and MetS should encourage dental and medical institutions to create guidelines for physicians, dentists, and patients to enhance prevention and management. The results of this study can help health providers inform patients about the components of MetS for better control of their MetS and consequently reduce the risk of dental caries. Furthermore, it will add value in the area of comprehensive care for patients, providers will be better informed about the relationship between MetS and dental caries, and, therefore, will advise their patients accordingly.

## Conclusions

To our knowledge, this is the first cross-sectional study using a US nationally representative sample that indicates a significant association between MetS and both a higher prevalence of untreated dental caries and elevated DMFT scores. Insulin resistance, abdominal obesity, and low HDL were the most significant MetS components related to the increase in the burden of dental caries. Future studies should aim to assess this association after controlling for important factors, including oral hygiene practices and the use of fluoridated toothpaste. Moreover, a properly conducted prospective cohort study would be important to establish causality between dental caries and MetS.
